# Transseptal Access to the Left Atrium: A Narrative Review of Techniques, Indications, and Device Innovations

**DOI:** 10.3390/life16071179

**Published:** 2026-07-16

**Authors:** Andrei Mihnea Rosu, Theodor Georgian Badea, Florentina Luminita Tomescu, Emanuel Stefan Radu, Maria-Daniela Tanasescu, Eduard George Cismas, Oana Andreea Popa

**Affiliations:** 1 Department of Cardiology, Prof. Dr. Agripa Ionescu Emergency Hospital, 077015 Balotesti, Romania; andrei-mihnea.rosu@drd.umfcd.ro (A.M.R.); radu.emanuel@dcti.ro (E.S.R.); popa.oana@dcti.ro (O.A.P.); 2 Department of Radiology, Prof. Dr. Agripa Ionescu Emergency Hospital, 077015 Balotesti, Romania; theodor.badea@umfcd.ro (T.G.B.); luminita.tomescu@umfcd.ro (F.L.T.); 3 Department of Radiology, Carol Davila University of Medicine and Pharmacy, 022328 Bucharest, Romania; 4 Department of Semiology, Emergency University Hospital, Carol Davila University of Medicine and Pharmacy, 022328 Bucharest, Romania; 5 Department of Cardiology, “Sf. Ioan” Emergency Clinical Hospital, 061344 Bucharest, Romania

**Keywords:** transseptal puncture, left atrial access, structural heart interventions, electrophysiology, radiofrequency-assisted puncture, atrial septoplasty, intracardiac imaging, mitral valve repair

## Abstract

Transseptal puncture (TSP) is a critical technique for accessing the left atrium in various structural and electrophysiological cardiac procedures. Originally introduced for diagnostic catheterization in the mid-20th century, it has evolved into a cornerstone of modern interventional cardiology. This article was designed as a targeted narrative review, rather than a systematic or comprehensive review, and synthesizes selected peer-reviewed evidence spanning 1955 to 2025, retrieved through a targeted literature search. We explore the anatomical foundations of TSP, its historical development, and modern refinements such as radiofrequency-assisted puncture, balloon septoplasty, and fluoroless or image-fusion-guided access. Clinical applications—including mitral valve interventions, left atrial appendage closure, and decompression during extracorporeal membrane oxygenation (ECMO)—are reviewed alongside safety considerations and complication management strategies. Advances in imaging modalities, including three-dimensional echocardiography and computed tomography, have enhanced precision and safety. Because of the narrative design, the review emphasizes clinical relevance, procedural applicability, and evidence synthesis without formal risk-of-bias scoring or quantitative evidence grading. Overall, TSP demonstrates a high success rate and low complication profile when performed with appropriate imaging and operator expertise. Ongoing innovation in technique and technology continues to expand its utility across cardiac disciplines.

## 1. Introduction

Accurate assessment of left heart hemodynamics is central to the diagnosis and management of structural heart disease. Early methods for accessing the left atrium were limited by complexity and procedural risk, particularly in critically ill patients or those with challenging anatomy. The introduction of transseptal puncture (TSP) offered a safer and more reproducible approach via femoral venous access. It enabled high-fidelity pressure measurements, contrast studies, and, in selected cases, advancement into the left ventricle or aorta [1,2]. Initial applications under fluoroscopic guidance demonstrated feasibility [2], and subsequent large series confirmed its safety and reliability [3]. Although RF-assisted, ICE-guided, and fusion-guided approaches have expanded the technical options for transseptal access, the classical sheath–dilator–needle technique remains the practical reference approach. The durability of needle-based transseptal access is reflected in the early Brockenbrough experience of 450 transseptal left heart catheterizations, with no reported mortality, accidental aortic puncture in three patients, and successful left ventricular catheterization in 143 of 151 attempted percutaneous studies [3].

Over the past two decades, TSP has become a cornerstone of left atrial access for both electrophysiological and structural interventions. In electrophysiology, it is essential for atrial fibrillation ablation and other left-sided procedures, where successful outcomes rely on safe and precise septal crossing [4,5]. In structural cardiology, TSP facilitates therapies such as mitral valve repair or replacement and left atrial appendage occlusion. It has also proven feasible in critically ill patients on mechanical circulatory support, where left atrial venting via extracorporeal membrane oxygenation (ECMO) circuits improves hemodynamic stability [6].

As procedural complexity and volumes increase, so does the reliance on TSP. Clinical guidelines and expert consensus documents now recognize it as fundamental in both atrial fibrillation management and thromboembolic prevention strategies [7]. However, expanding indications have introduced anatomical and technical challenges, especially with the rise in transcatheter mitral and left atrial appendage interventions. Successful septal access increasingly depends not only on operator skill but also on detailed knowledge of interatrial septal anatomy, its variation across patients, and its spatial relationship to surrounding cardiac structures [8]. In contemporary structural heart practice, TSP is still described as a stepwise sheath–dilator–needle procedure, with RF-assisted systems used as adjunctive escalation tools in selected resistant septa rather than as universal first-line replacements [8]. The use of advanced imaging—including intracardiac echocardiography, transesophageal echocardiography, and computed tomography—has further improved safety and precision during septal puncture.

This review examines contemporary transseptal puncture techniques, with emphasis on their anatomical basis, technological evolution, and current clinical relevance. We highlight innovations in imaging, devices, and procedural strategies that have expanded TSP’s role across diverse cardiovascular interventions. The review is structured to address anatomical considerations, historical development, modern techniques, clinical applications, and safety management.

## 2. Materials and Methods

This narrative review provides an integrated synthesis of the anatomical, procedural, and clinical evolution of TSP from its early origins to present-day innovations. A targeted literature search was performed using PubMed, Web of Science, and Google Scholar, complemented by manual screening of reference lists from seminal articles and expert consensus statements. The search spanned publications from January 1955 through July 2025, aiming to capture both foundational developments and recent advancements. The final literature query was completed on 15 August 2025.

Search terms included combinations of: “transseptal puncture”, “interatrial septum”, “left atrial access”, “Brockenbrough needle”, “radiofrequency-assisted puncture”, “septoplasty”, “fusion imaging”, “structural heart interventions”, “iatrogenic atrial septal defect”, “LAAC”, “TMVR”, “ECMO”, and “MitraClip access.” Additional keywords such as “Cope technique,” “Ross study,” and “pediatric transseptal catheterization” were included to identify key historical contributions.

Inclusion criteria: peer-reviewed original studies, clinical trials, case series, review articles, expert consensus documents, and relevant guidelines published between 1955 and 2025 in English, reporting on human TSP procedures for diagnostic or interventional purposes. Given the historical nature of early TSP development, selected foundational experimental studies were also considered when they provided essential technical or historical context.

Exclusion criteria: non-English publications; pediatric-only studies unless they contributed foundational insights; conference abstracts or editorials lacking primary data; and animal studies, unless of historical significance (e.g., early canine experiments by Ross). Studies were prioritized when they contributed directly to one of the predefined thematic domains of the review, rather than on the basis of formal quantitative ranking.

Out of 312 articles identified, studies and expert documents were selected following full-text screening and relevance assessment, based on their relevance to the predefined thematic domains of the review. Two reviewers independently conducted the screening, resolving any discrepancies by discussion. This selection process was intended to support a focused clinical and technical synthesis, not an exhaustive systematic evidence map.

The final selection of studies addressed five key domains:

1. Historical and technical evolution;
2. Imaging and anatomical considerations;
3. Device and energy-based innovations;
4. Clinical applications;
5. Safety, risk management, and complication strategies.

Given the narrative design of this review, no meta-analysis, quality assessment tools, or risk-of-bias scoring were applied. Included studies were synthesized qualitatively, with an emphasis on clinical applicability, procedural advancements, safety outcomes, and implications for future research. Accordingly, the findings should be interpreted as a narrative synthesis of selected evidence rather than as a quantitatively graded assessment of the entire literature.

**Figure 1:**
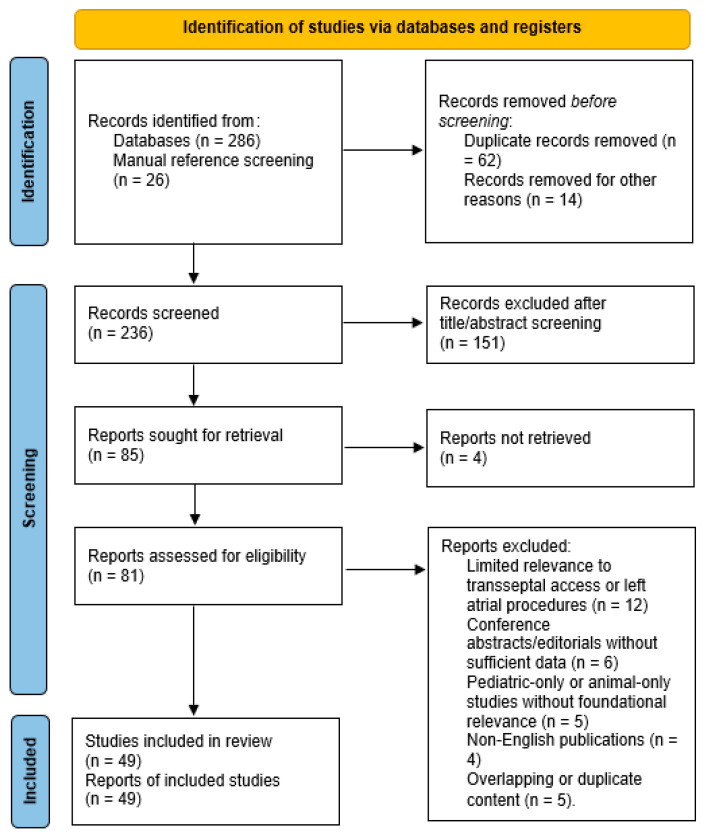
Simplified literature selection flow diagram for the targeted narrative review.

The literature selection process is summarized in a simplified PRISMA-style flow diagram, used here only to improve transparency of the targeted narrative search and not to imply systematic-review methodology (Figure 1).

## 3. Evolution of Transseptal Puncture Techniques

TSP was first developed in the late 1950s as a novel method to access the left atrium through a percutaneous venous approach. In a landmark 1959 study, Ross introduced the technique by performing left atrial catheterization in canine models. He used a retractable needle inserted through a modified Cournand catheter to traverse the interatrial septum under fluoroscopic guidance. This approach allowed direct pressure measurement, selective contrast injection, and, in some cases, catheter advancement into the left ventricle or aorta. The method offered a safer alternative for patients who were unsuitable for transbronchial or suprasternal access routes [1]. Building on this work, Brockenbrough, Braunwald, and Ross published a fundamental 1962 study describing 450 successful transseptal catheterizations in humans. They introduced a refined technique using a 17-gauge thin-walled needle (later known as the Brockenbrough needle) and a pre-shaped radiopaque catheter capable of advancing across the mitral valve. Their percutaneous approach, based on the Seldinger technique, proved safe, reproducible, and adaptable across a wide range of patient anatomies [3], laying the groundwork for widespread clinical adoption.

Subsequent innovations included the development of the Mullins sheath—a dilator and sheath system designed to work in tandem with the Brockenbrough needle, providing a smooth, coaxial transition through the septum [9]. Today, although “Brockenbrough needle” and “Mullins sheath” are often used generically, manufacturers offer various tailored systems, particularly for electrophysiological procedures such as atrial fibrillation ablation [10].

As clinical use expanded into more complex interventions, the limitations of purely mechanical puncture became evident—especially in patients with fibrotic, aneurysmal, or surgically altered septa. These challenges prompted the introduction of radiofrequency (RF)-assisted transseptal access, which applies brief bursts of RF energy through the needle tip to reduce resistance and minimize the need for mechanical force. Performed under fluoroscopic and echocardiographic guidance, this technique improved procedural control and safety, particularly in patients with abnormal septal anatomy or those on anticoagulation [11]. Prospective studies confirmed its utility in difficult cases, reporting consistent first-attempt success and no procedural complications [12].

Despite these advances, access can remain difficult in patients with prior septal interventions, thickened tissue, or multiple previous ablations. In such cases, atrial balloon septoplasty (ABS) has emerged as a valuable adjunct technique. This approach involves the inflation of a noncompliant balloon across the puncture site to enlarge the tract and facilitate sheath advancement. Performed over a stiff guidewire placed in a left pulmonary vein, ABS has demonstrated high success rates and a favorable safety profile in patients with septal scarring, occluder devices, or surgical patches [13,14].

These innovations—spanning over six decades—reflect a steady refinement of TSP from a diagnostic maneuver into a versatile and adaptable tool for modern cardiac interventions. The evolution of access strategies continues to address anatomical barriers and procedural risks, forming the foundation for further device integration and image-guided approaches.

## 4. Anatomical and Imaging Considerations

Successful TSP depends on precise anatomical localization to minimize the risk of complications, especially in patients with atypical cardiac anatomy or limited imaging landmarks. Fluoroscopic guidance alone can be misleading, as misinterpretation of structures has occasionally resulted in inadvertent puncture of adjacent tissues, such as the aortic root. In rare cases, sheath advancement into the non-coronary aortic cusp has necessitated percutaneous repair [15].

To reduce such risks, multislice computed tomography (MSCT) has emerged as a valuable pre-procedural tool alongside traditional echocardiography. MSCT can delineate key structures like the fossa ovalis and simulate optimal catheter angulation using 3D reconstruction software (RecFusion Pro 2.2.0). When overlaid onto fluoroscopic images, these reconstructions enhance real-time spatial orientation and guide safer catheter manipulation during left atrial interventions such as mitral valve repair or left atrial appendage closure [16].

In high-risk patients—such as those with left atrial thrombus or a history of embolic events—TSP carries a heightened risk of cerebral embolism. In these cases, cerebral protection systems (e.g., filter-based devices placed in supra-aortic vessels) can capture embolic debris without disrupting catheter function. This strategy has proven effective during complex procedures, including combined mitral valvuloplasty and appendage closure [17].

Fusion imaging technologies further enhance procedural control. For example, integration of transesophageal echocardiography (TEE) with fluoroscopy allows synchronized visualization of the fossa ovalis and adjacent structures. This method improves puncture site selection and spatial orientation, reducing procedure time and complications, particularly during repeat or anatomically complex interventions [18].

Three-dimensional (3D) TEE, when fused with fluoroscopy, provides personalized guidance by accounting for variations in atrial anatomy. It can optimize puncture angle and trajectory, especially in patients with distorted or previously treated septa [19]. Similarly, overlaying 3D cardiac CT images onto fluoroscopic views aids in pre-procedural planning for left atrial appendage closure by helping to localize the appendage’s depth and orientation. This method can minimize fluoroscopy time, reduce repositioning, and improve device deployment accuracy [20].

In addition to procedural guidance, MSCT has also been used to map and visualize fluoroscopic landmarks of key cardiac structures—including the pulmonary veins, mitral annulus, and left atrial appendage. Simulating ideal C-arm angles and catheter paths may enhance reproducibility and safety across a broad spectrum of left atrial procedures [21].

Beyond localization of the fossa ovalis, the transseptal puncture site should be tailored to the planned intervention. In routine electrophysiology procedures, a central or slightly posterior puncture is often sufficient for left atrial access. In contrast, structural mitral interventions require greater attention to puncture location because it directly affects catheter trajectory, working space, and device alignment. For TEER (MitraClip) and TMVR, a posterior and relatively inferior puncture is generally preferred, as it may facilitate alignment with the mitral valve and reduce the need for excessive catheter deflection.

The inferoposterior region should be regarded as a procedural target rather than a fixed anatomical requirement. The optimal puncture site depends on multiple factors, including left atrial dimensions, septal anatomy, mitral valve pathology, device characteristics, and operator strategy. An anterior puncture may result in an unfavorable approach to the mitral valve, whereas an excessively superior puncture can impair catheter maneuverability. Conversely, a puncture that is too low may limit the working height required for stable device manipulation. Accordingly, puncture-site selection should consider both septal location and its spatial relationship to the mitral annular plane.

Tenting height is a key parameter in TEER planning and is defined as the distance between the tented interatrial septum and the mitral annular plane, typically assessed by intraprocedural TEE before septal crossing. Adequate tenting height provides sufficient working space for device steering, leaflet alignment, and grasping. Insufficient height may restrict maneuverability, whereas excessive height or suboptimal orientation may necessitate greater catheter deflection to reach the target leaflet segments. Therefore, TEER generally requires a posterior puncture with adequate height to balance access and device control. Similar considerations apply to TMVR, although greater emphasis is placed on delivery-system coaxiality and accommodation of larger device profiles.

## 5. Imaging Guidance for Transseptal Access

In contemporary practice, the classical transseptal puncture technique remains the foundation for left atrial access and relies on the combined use of fluoroscopic and echocardiographic guidance to ensure accurate localization of the fossa ovalis and minimize procedural risk. Following femoral venous access and advancement of the transseptal sheath–dilator assembly into the superior vena cava, the Brockenbrough needle is positioned and withdrawn under controlled tension toward the interatrial septum.

Imaging guidance during TSP has two complementary purposes: to confirm safe engagement of the fossa ovalis and to adapt the puncture site to the intended left atrial target. The relative value of each modality differs according to the procedure, patient anatomy, available equipment, and operator experience. Fluoroscopy remains the basic spatial framework for many procedures, while TEE, ICE, MSCT, fusion imaging, and electroanatomic mapping provide additional anatomical or real-time information in selected settings.

### 5.1. Fluoroscopy and Fluoroscopic Landmarks

**Figure 2:**
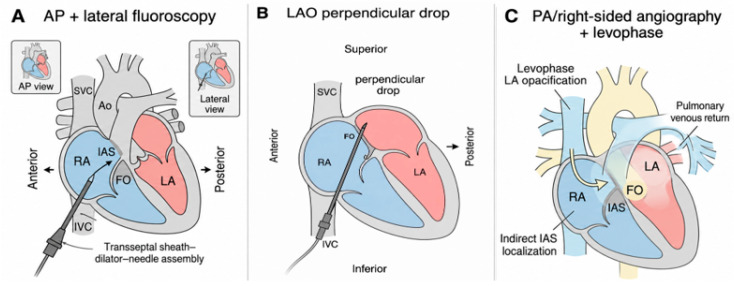
Classical fluoroscopic approaches to transseptal puncture. In all panels, the transseptal sheath–dilator–needle assembly is advanced from the venous system into the right atrium, positioned against the fossa ovalis, and directed from the right atrium toward the left atrium. (**A**) AP and lateral fluoroscopy help define the general cranio-caudal, mediolateral, and anterior–posterior relationship of the transseptal sheath–dilator–needle assembly to the interatrial septum and adjacent structures. (**B**) In the LAO projection, controlled pull-down from the superior vena cava toward the fossa ovalis allows recognition of perpendicular septal engagement from the right atrial side; septal tenting and puncture are directed from RA to LA (**C**) PA or right-sided angiography with levophase left atrial opacification can indirectly delineate the left atrium and interatrial septal region when standard fluoroscopic landmarks are unclear. Ao, aorta; AP, anteroposterior; FO, fossa ovalis; IAS, interatrial septum; IVC, inferior vena cava; LA, left atrium; LAO, left anterior oblique; PA, pulmonary artery; RA, right atrium; SVC, superior vena cava. 2Fluoroscopy therefore provides the initial spatial framework for septal orientation. In the right anterior oblique (RAO) projection, the operator assesses the anterior–posterior position of the transseptal assembly, facilitating discrimination between the septum and adjacent structures such as the aortic root. The left anterior oblique (LAO) view complements this by defining the superior–inferior axis and confirming appropriate septal engagement. Characteristic septal “tenting” under gentle pressure serves as an additional fluoroscopic indicator of correct positioning prior to puncture.

Several classical fluoroscopic approaches remain relevant for understanding TSP orientation, even though contemporary procedures often combine fluoroscopy with TEE, ICE, CT-based planning, or fusion imaging. These approaches include AP and lateral fluoroscopy, LAO-guided pull-down with perpendicular septal engagement, and angiographic levophase delineation of the left atrium and interatrial septal region. In the AP–lateral method, the anteroposterior projection provides the general cranio-caudal and mediolateral position of the transseptal assembly, while the lateral projection helps assess its anterior–posterior relationship to the interatrial septum, left atrium, and aortic root. A second approach uses LAO fluoroscopy during the controlled pull-down from the superior vena cava toward the fossa ovalis; the so-called perpendicular drop helps the operator recognize septal engagement by following the descent and orientation of the sheath–dilator–needle assembly relative to the septal plane. A third classical method uses pulmonary artery or right-sided chamber angiography with levophase opacification, allowing indirect delineation of the left atrium and interatrial septal region when standard fluoroscopic landmarks are unclear [8] (Figure 2).

**Figure 3:**
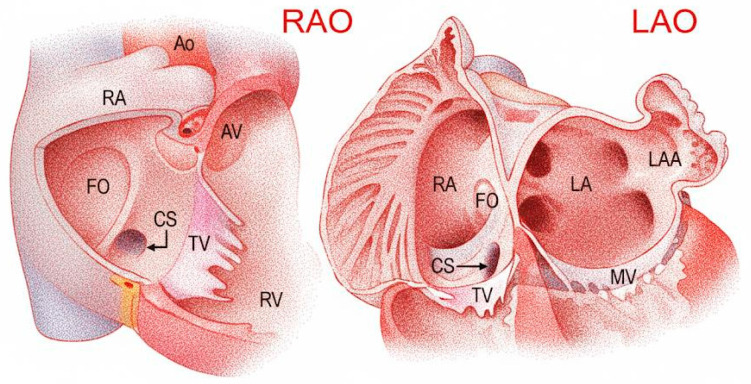
Angiographic RAO and LAO projections illustrating the anatomical landmarks used to define anterior–posterior and superior–inferior orientation during transseptal puncture. RAO: Right Anterior Oblique; LAO: Left Anterior Oblique; RA: right Atrium, LA: Left Atrium, LAA: Left Atrial Append-age; MV: Mitral Valve; TV: tricuspid valve; CS: coronary sinus; RV: right ventricle; AV: aortic valve; Ao: Aorta.

The main advantage of fluoroscopy is its availability and familiarity. It allows continuous tracking of the sheath–dilator–needle assembly during superior vena cava positioning, pull-down, septal engagement, and crossing. However, fluoroscopy provides only indirect anatomical information and cannot directly visualize septal thickness, the fossa ovalis, or the relationship between the needle tip and adjacent soft-tissue structures. For this reason, fluoroscopic landmarks are often supplemented by pressure tracing, contrast injection, or echocardiographic imaging in complex anatomy or structural interventions. MSCT-based anatomical studies have also helped clarify how left-sided structures such as the pulmonary veins, mitral annulus, left atrial appendage, and aortic root project under fluoroscopy, improving anatomical interpretation during transcatheter procedures [21] (Figure 3).

### 5.2. Transesophageal Echocardiography

However, fluoroscopy alone cannot reliably define the optimal puncture height or depth within the septum. For this reason, transesophageal echocardiography (TEE) plays a central role in modern TSP, with the echocardiographer providing real-time guidance in multiple planes. The bicaval view allows precise differentiation between superior versus inferior septal positioning and is essential for avoiding excessively high or low puncture sites. The short-axis view at the level of the aortic valve enables assessment of anterior versus posterior orientation, ensuring safe distance from the aortic root. Finally, the four-chamber view confirms the overall trajectory toward the left atrium and assists in fine-tuning puncture depth (high versus low), particularly in procedures requiring specific coaxial alignment.

TEE is particularly useful in structural heart procedures, where the puncture site determines not only safety but also device trajectory. In transcatheter edge-to-edge mitral repair, transcatheter mitral valve interventions, left atrial appendage closure, and left atrial decompression during VA-ECMO, TEE provides direct visualization of septal tenting, puncture height, adjacent structures, and early complications. Its limitations are mainly practical: the need for an experienced echocardiographer, patient tolerance issues, frequent use of deep sedation or general anesthesia, and the need for continuous coordination between the operator and the imaging specialist.

**Figure 4:**
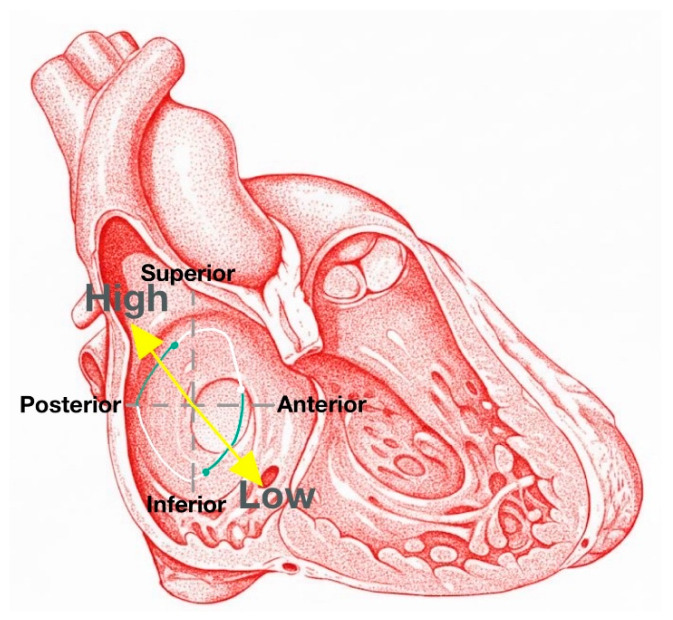
Integration of RAO fluoroscopic view with echocardiographic guidance to define anterior–posterior orientation during transseptal puncture.

**Figure 5:**
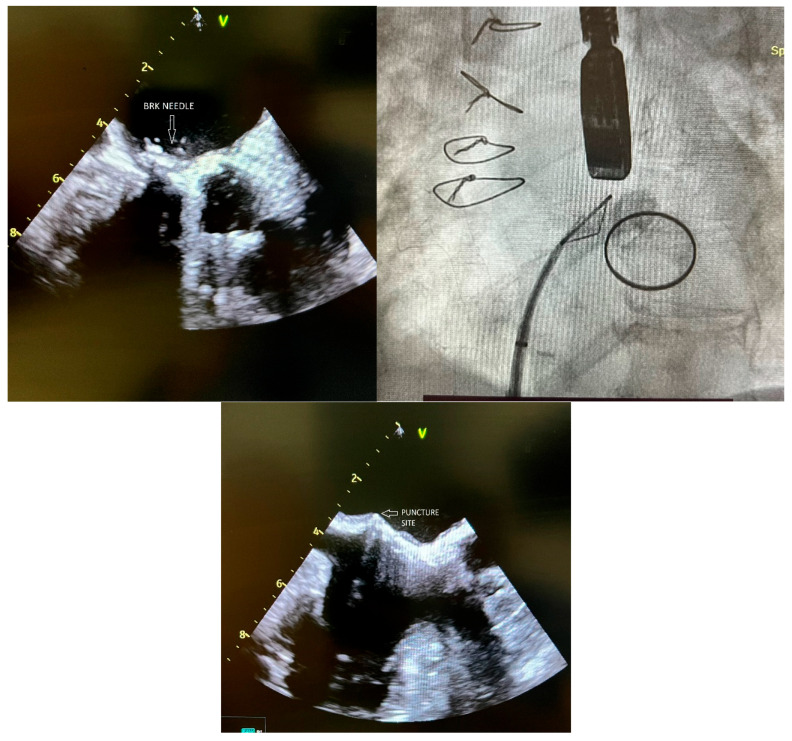
Case-based illustration showing the transseptal system in a 30° left anterior oblique (LAO) projection in a patient with double prosthetic valves (aortic and mitral). Echocardiographic guidance was decisive for safe transseptal puncture, with visualization provided by the bicaval view and the short-axis view at the level of the ascending aorta.

This continuous interaction between operator and echocardiographer is critical, especially during interventions where the puncture site directly influences procedural success, such as transcatheter mitral valve repair or left atrial appendage closure (Figure 4 and Figure 5).

### 5.3. Intracardiac Echocardiography

Intracardiac echocardiography (ICE) deserves separate consideration because its role differs from both fluoroscopy and TEE. ICE provides real-time intracardiac imaging from a venous approach and can directly visualize the interatrial septum, the fossa ovalis, the transseptal sheath, needle contact, and septal tenting. ICE is described as a practical tool for electrophysiologists because it permits direct endocardial visualization and precise localization of the transseptal needle and sheath against the interatrial septum, This is especially useful when the septum is thickened, floppy, aneurysmal, previously sutured, or crossed by an occluder device, and when right atrial structures such as a prominent Eustachian ridge or Chiari network complicate catheter manipulation [22].

From a workflow perspective, ICE is particularly relevant in electrophysiology laboratories. It can be performed through femoral venous access under local anesthesia, avoids esophageal instrumentation, and may reduce the need for general anesthesia compared with TEE in selected cases. The Chinese expert consensus on ICE recommends ICE guidance during TSP, especially in patients with abnormal atrial size, abnormal septal anatomy, congenital cardiac anomalies, or distorted thoracic anatomy, and also supports its use during AF ablation to reduce radiation exposure. The same consensus notes that ICE can visualize the fossa ovalis, guide puncture-site selection, show the tenting sign, and confirm left atrial entry by saline microbubble appearance after needle crossing [23].

ICE also supports low-fluoroscopy and zero-fluoroscopy strategies when combined with three-dimensional electroanatomic mapping. Real-time ICE guidance can allow zero-X-ray TSP in selected cases, although this approach is generally reserved for experienced operators, and conventional fluoroscopy may still be combined with ICE when needed [23]. Modified ICE-guided workflows using ablation catheter guidance have been associated with fewer puncture attempts and shorter puncture duration, without compromising procedural success, puncture-site accuracy, or procedural safety [24]. These findings support the growing procedural role of ICE in contemporary EP practice, while also indicating that ICE-guided workflows remain technique-dependent and require specific training.

The limitations of ICE should also be acknowledged. It requires an additional venous catheter, increases procedural cost, and depends on operator familiarity with ICE views and image-plane orientation. In contrast to TEE, ICE may offer fewer standard views, although it provides near-field intracardiac imaging and can be controlled directly by the operator. Therefore, ICE should be presented as complementary to fluoroscopy and TEE rather than as a universal replacement for either modality.

### 5.4. Multislice Computed Tomography and Preprocedural Planning

MSCT is not usually the primary real-time imaging tool for TSP, but it is highly useful for preprocedural planning. It can define the spatial relationships among the fossa ovalis, pulmonary veins, left atrial appendage, mitral annulus, and aortic root. These relationships are particularly important in patients with distorted anatomy, prior septal intervention, planned left atrial appendage closure, or transcatheter mitral procedures. Thériault-Lauzier et al. used MSCT to clarify the fluoroscopic anatomy of left-sided heart structures, supporting a more accurate interpretation of catheter position during transcatheter interventions [25].

In left atrial appendage closure, integration of fluoroscopy with three-dimensional CT datasets has been used to improve spatial orientation and device guidance [25]. Similarly, CT-optimized fluoroscopic guidance has been applied to transcatheter mitral therapies, where the relationship between the transseptal trajectory and the mitral annular plane directly affects catheter alignment and device delivery [19]. The main limitations of MSCT are radiation exposure, contrast administration, and the static nature of preprocedural images. These limitations can be partly offset when CT datasets are incorporated into live fluoroscopic workflows.

### 5.5. Fusion Imaging, Electroanatomic Mapping, and Fluoroless Guidance

Fusion imaging combines live procedural imaging with anatomical information obtained from TEE or CT. TEE–fluoroscopy fusion can improve visualization of the fossa ovalis and adjacent structures during puncture, while CT–fluoroscopy overlay may help align the transseptal trajectory with target structures such as the left atrial appendage or mitral annulus. These methods are most useful in structural heart interventions, repeat procedures, and anatomically complex cases, but they require dedicated software, institutional infrastructure, and experienced operators.

Electroanatomic mapping systems provide another route to reduce fluoroscopy, particularly in EP procedures. When combined with ICE, these systems allow the operator to integrate septal anatomy, catheter position, and the intended puncture site into a three-dimensional procedural map. The recent ICE-guided TSP literature supports this direction, but also indicates that these approaches are not purely equipment-driven; they depend on operator experience, reproducible workflows, and careful recognition of septal anatomy [23,24].

With increasing procedural complexity, device selection plays a critical role in procedural success. Table 1 offers a practical comparison of available transseptal tools and innovations.

Nevertheless, these modalities should not be considered interchangeable. Fluoroscopy remains the practical baseline for many procedures; TEE provides robust soft-tissue guidance for structural heart interventions; ICE has a growing role in EP workflows and selected structural procedures; MSCT supports anatomical planning; and fusion or electroanatomic systems may improve precision in complex or radiation-reduction strategies. The imaging strategy should therefore be individualized according to the intervention, septal anatomy, device trajectory, patient risk, and institutional expertise.

**Figure 6:**
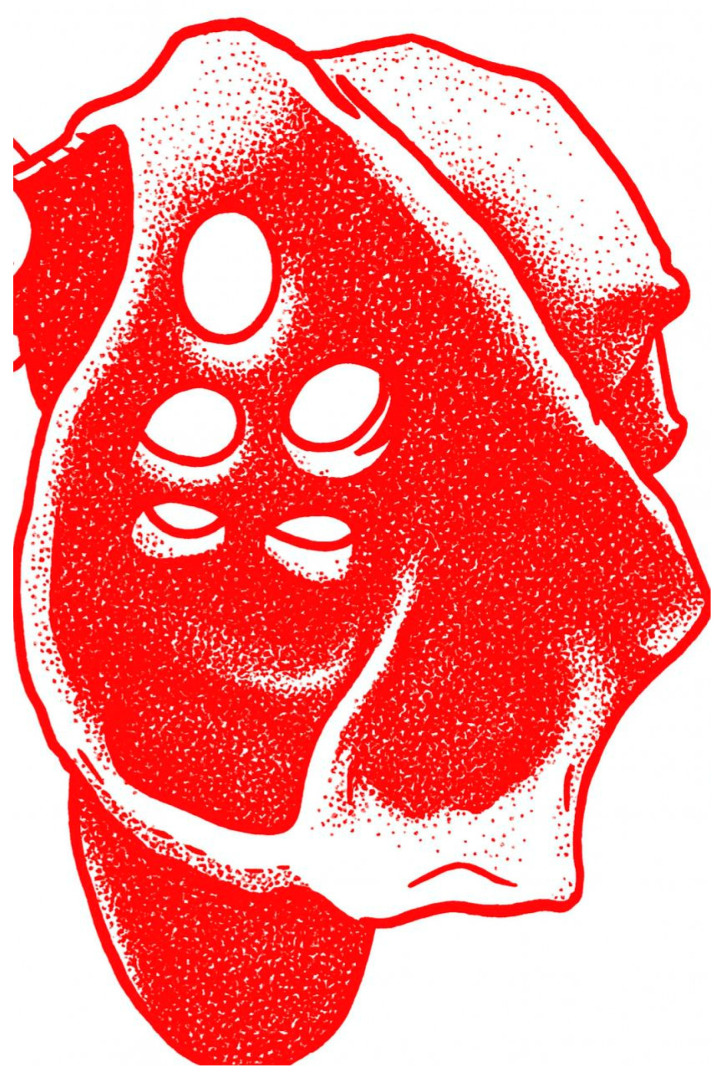
Left atrial perspective of the interatrial septum showing the five possible transseptal puncture sites according to procedural orientation. The figure illustrates the central fossa ovalis region and the four peripheral quadrants—superoanterior, inferoanterior, superoposterior, and inferoposterior—which are selected according to the intended left atrial target and device trajectory. The anterior–posterior, superior–inferior, and medial–lateral orientations should be interpreted together during puncture planning, particularly in structural heart procedures where puncture height and posterior positioning may influence catheter maneuverability and coaxial alignment.

By integrating fluoroscopic landmarks with systematic echocardiographic confirmation across the bicaval, aortic short-axis, and four-chamber views, the classical TSP technique achieves a high degree of reproducibility, safety, and adaptability across a wide range of anatomical scenarios (Figure 6).

**Figure 7:**
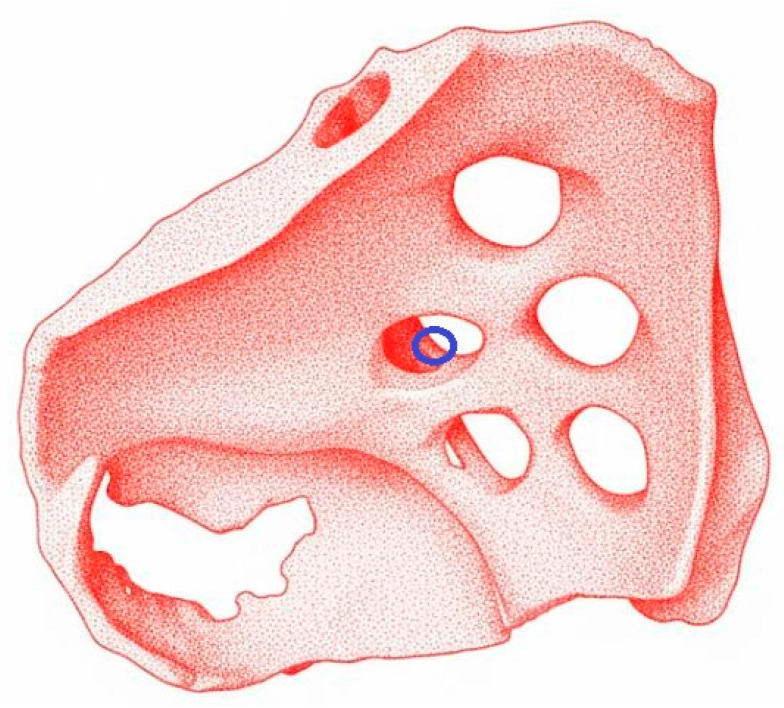
Right atrial perspective of the interatrial septum showing the five possible transseptal puncture sites according to procedural orientation, with infero-posterior zone marked (blue circled). The central fossa ovalis region and the superoanterior, inferoanterior, superoposterior, and inferoposterior quadrants are interpreted in relation to the anterior–posterior, superior–inferior, and medial–lateral axes. The inferoposterior zone is marked with a blue circle, as this region is often relevant for procedures requiring a posterior and inferior transseptal trajectory, particularly selected mitral interventions where puncture height and device alignment influence procedural maneuverability.

Clinically, the *fossa ovalis* represents the target region for transseptal puncture due to its status as the thinnest portion of the interatrial septum and its relatively consistent anatomic relationships. From an anatomic standpoint, this area can be conceptualized in a segmented fashion to guide optimal puncture site selection. Classically, the fossa ovalis has been divided into four quadrants—superior–anterior, inferior–anterior, superior–posterior, and inferior–posterior (Figure 4)—corresponding to its spatial orientation relative to nearby structures such as the superior vena cava, coronary sinus, and aortic root; variations in septal shape and atrial geometry influence these designations and may affect access strategy (Figure 7).

Beyond these quadrants, many operators also describe a central or mid-fossa location, representing the geometric center of the depression and often considered the optimal puncture point in standard anatomy. In more complex procedures, such as pulmonary vein isolation or structural interventions, site selection may be tailored based on therapeutic goals: *infero-posterior* placements can facilitate access to the inferior pulmonary veins and posterior structures, whereas *supero-anterior* approaches may favor anterior structures but carry a theoretical risk of proximity to the aortic root if not confirmed with imaging.

Thus, in practice, a spectrum of five conceptual puncture zones—central, superior–anterior, inferior–anterior, superior–posterior, and inferior–posterior—is recognized. Accurate localization within this framework, informed by multimodality imaging, enhances procedural efficacy and minimizes complications by aligning the transseptal entry point with the intended left atrial target and catheter trajectory.

## 6. Modern Approaches and Device Innovations

TSP has undergone substantial technological refinement in response to anatomical barriers and procedural challenges. One early obstacle involved patients with prior atrial septal repairs—such as surgical patches or baffles—where conventional needle access often failed due to fibrotic or prosthetic tissue. To address this, new strategies emerged, including the use of steerable sheaths and reinforced catheter assemblies capable of traversing resistant septa. In some cases, multiple punctures were necessary to identify a viable entry point into the left atrium [15].

A major innovation was the adaptation of standard coronary angioplasty guidewires for electrosurgical use. By connecting the proximal end of a stiff wire to an electrocautery unit, clinicians could apply controlled bursts of RF energy through the tip, enabling puncture of dense or previously ablated septal tissue with minimal force [26]. This off-label technique demonstrated consistent success and maintained procedural safety.

Purpose-built RF transseptal systems were later developed to standardize this approach. These devices feature RF-enabled needle tips that deliver low-energy pulses to facilitate septal crossing, even in cases with thickened, aneurysmal, or previously scarred tissue. Compared to conventional Brockenbrough needles, RF systems improve first-pass success and reduce mechanical resistance while preserving safety [27].

Other adaptations have made RF technology more accessible. For instance, connecting a standard Brockenbrough needle to a conventional RF ablation generator allows for energy-assisted puncture using existing equipment. This method has proven effective in difficult anatomies without adding procedural complexity [11].

Mechanical innovations have also emerged. The SafeSept guidewire, with its ultra-sharp tip and flexible shaft, allows passage through the septum with minimal force and reduced risk of perforation. It integrates with standard sheaths and dilators, making it a practical solution for difficult cases [28].

In addition to tool development, imaging innovations have enhanced precision. CT-based fluoroscopic guidance, which overlays 3D anatomical reconstructions onto live fluoroscopy, helps align catheters with targets like the mitral annulus or left atrial appendage. This reduces the need for repositioning and shortens procedure time [15].

Even in patients with previously implanted septal occluder devices, transseptal access can be achieved. Modified needle techniques combined with progressive balloon dilation allow for safe passage of large-caliber sheaths—such as those used in MitraClip implantation—by targeting accessible regions of the device [29]. Similarly, electroanatomic mapping systems can guide puncture without fluoroscopy or echocardiographic imaging. Voltage mapping reliably identifies the fossa ovalis, enabling radiation-free and contrast-free TSP in experienced hands [30]. Transesophageal echocardiography (TEE) may also be used to guide puncture across occluder devices, confirming feasibility and safety when imaging guidance is optimized [31].

Recent innovations focus on further simplifying the procedure and reducing dependence on fluoroscopy. Three-dimensional electroanatomic mapping systems now enable entirely fluoroless TSP by integrating catheter tracking and septal geometry in real time. These systems have demonstrated safe and effective use in routine ablation workflows [32].

One notable advancement is the VersaCross RF system, which integrates an RF puncture wire, sheath, and dilator into a single platform. Comparative studies show that it reduces puncture time and facilitates sheath advancement, without increasing complications [33].

With increasing procedural complexity, device selection plays a critical role in procedural success. Table 2 offers a practical comparison of available transseptal tools and innovations.

## 7. Clinical Indications and Applications

TSP has become a foundational technique in both structural and electrophysiological cardiac procedures requiring left atrial access. While it was originally developed for diagnostic purposes, its clinical utility now spans a wide range of interventions, including atrial fibrillation ablation, left atrial appendage occlusion, and transcatheter mitral valve repair or replacement.

In electrophysiology, TSP is routinely performed for pulmonary vein isolation and other left-sided arrhythmia ablations. A large international survey confirmed its widespread use, often guided by fluoroscopy and pressure monitoring alone [34]. TSP has also been applied successfully in high-risk patients—such as those with spontaneous echo contrast or known left atrial thrombus—when guided by advanced imaging and cerebral protection devices [35].

The technique remains indispensable in patients with complex atrial anatomy due to prior surgery or congenital anomalies. Transseptal access through atrial baffles, surgical patches, or conduits has been shown to be feasible using modified needle trajectories and dedicated sheath support [22].

In selected patients, a naturally patent foramen ovale may provide an alternative route for left atrial access. ICE-guided procedural descriptions show that a guidewire can be advanced through a PFO slit and that ICE can define the PFO tunnel, residual atrial-level shunting, and adjacent septal structures [23]. However, PFO-based crossing should be considered opportunistic rather than routine, because the tunnel may not provide the puncture height, posterior orientation, or catheter trajectory required for procedures such as TEER, TMVR, or LAAC. When this route is considered, TEE or ICE guidance is useful to confirm the tunnel course, wire position, and suitability for the intended left atrial target.

In the structural heart field, TSP is required for procedures such as MitraClip implantation, balloon mitral valvuloplasty, and left atrial appendage closure. National registry data demonstrate its growing use across diverse patient populations and increasing procedural volumes [6]. However, anatomical challenges, such as fibrotic septa or previously closed defects, can complicate access. In these cases, bailout techniques like atrial balloon septoplasty have been employed. This involves inflating a noncompliant coronary balloon across the septum to dilate scarred or resistant tissue, allowing safe sheath advancement. The technique has shown high technical success with minimal complications [13].

TSP is also critical for advanced transcatheter mitral valve interventions, including valve-in-valve (ViV), valve-in-ring (ViR), and valve-in-mitral annular calcification (ViMAC) procedures. These require precise septal puncture to achieve coaxial alignment of the delivery system with the mitral valve plane [36].

In select patients with persistent left atrial thrombus—traditionally a contraindication to device-based interventions—a thrombus-trapping approach has enabled safe TSP and device delivery. This technique avoids direct contact with the thrombus by using modified delivery strategies and dedicated occlusion devices. It provides a viable treatment pathway for high-risk patients who are not candidates for long-term anticoagulation [37].

Because the optimal imaging strategy differs across procedures, imaging selection should be linked to the intended left atrial target, device trajectory, septal anatomy, and procedural risk. In routine electrophysiology procedures, fluoroscopy with pressure monitoring may be sufficient in experienced centers, although ICE and electroanatomic mapping are increasingly used in low- or zero-fluoroscopy workflows. In LAAC, TEER/MitraClip, and TMVR, imaging has a stronger planning role because puncture height, anterior–posterior orientation, and device coaxiality directly influence procedural feasibility. In redo TSP, prior septal repair, scarred septa, or occluder-device crossing, multimodality imaging and adjunctive access tools may be required to identify a safe residual septal window. A procedure-specific summary of imaging guidance strategies is provided in Table 3.

**Table 3:** Procedure-specific imaging guidance strategies for transseptal access.

Procedure/Indication	Imaging Strategy	TSP-Specific Objective	Key Procedural Note
AF ablation/left-sided EP procedures	Fluoroscopy ± pressure monitoring; ICE/EAM in low-fluoroscopy workflows	Safe fossa ovalis crossing and confirmation of LA entry	ICE/EAM may be useful in redo procedures or radiation-reduction protocols
LAAC	TEE or ICE with fluoroscopy; CT or fusion imaging when available	Select puncture site according to LAA orientation and device coaxiality	Thrombus assessment and device alignment guide imaging choice
TEER/MitraClip	TEE-guided TSP with fluoroscopy; CT/fusion imaging in selected anatomy	Define puncture height and anterior–posterior orientation for leaflet access	Tenting height and puncture location directly influence device maneuverability
TMVR/ViV/ViR/ViMAC	CT planning with intraprocedural TEE and fluoroscopy	Align transseptal trajectory with the mitral annular plane and delivery system	Large-bore delivery requires careful trajectory planning
VA-ECMO left atrial decompression	TEE with fluoroscopy	Confirm safe LA entry and decompression catheter position	Hemodynamic instability and anticoagulation require real-time monitoring
Redo TSP/prior septal closure/scarred septum	TEE or ICE with fluoroscopy; CT/fusion imaging and adjunctive tools as needed	Identify safe residual septal tissue and guide difficult crossing	RF-assisted puncture or balloon septoplasty may be required

Abbreviations: AF, atrial fibrillation; CT, computed tomography; EP, electrophysiology; ICE, intracardiac echocardiography; LA, left atrium; LAAC, left atrial appendage closure; LAA, left atrial appendage; RF, radiofrequency; TEE, transesophageal echocardiography; TEER, transcatheter edge-to-edge repair; TMVR, transcatheter mitral valve replacement; TSP, transseptal puncture; VA-ECMO, venoarterial extracorporeal membrane oxygenation; ViMAC, valve-in-mitral annular calcification; ViR, valve-in-ring; ViV, valve-in-valve. An overview of key clinical scenarios for TSP—including associated procedures, patient subgroups, and adjunctive techniques—is presented in Table 4.

**Table 4:** Clinical Applications of Transseptal Puncture Across Interventional Settings.

Clinical Application	Patient Setting	Associated Procedures	Adjunct Techniques	Notes
Pulmonary vein isolation (AF ablation)	Electrophysiology (EP)	AF ablation, left-sided arrhythmias	Fluoroscopy, pressure monitoring	Standard in EP labs; routinely performed [32]
Left atrial appendage occlusion	Structural/High-risk	Watchman™, Amulet™ implants	TEE, cerebral protection	Used in patients with thrombus or stroke risk [29,31]
Transcatheter mitral valve repair/replacement	Structural Heart	MitraClip, TMVR, ViV, ViR, ViMAC	TEE, preprocedural CT	Requires precise septal puncture for coaxiality [6,30]
Balloon mitral valvuloplasty	Rheumatic or pregnant patients	Percutaneous mitral dilation	TEE, low-radiation protocol	Feasible and safe in pregnancy [34]
Anatomical challenges (e.g., baffles, patches)	Congenital or post-surgical	Customized TSP	Needle redirection, sheath support	Safe access achievable with modified technique [22]
Bailout access in resistant septa	All settings	Balloon septoplasty	Coronary balloon inflation	High success in failed standard TSP cases [13]
LA thrombus with closure indication	High stroke risk, anticoagulation contraindicated	Thrombus-trapping LAAC (TTP-LAAC)	No-thrombus manipulation strategy	Safe and successful in selected patients [31]

Abbreviations: AF, atrial fibrillation; CT, computed tomography; EP, electrophysiology; LA, left atrium; LAAC, left atrial appendage closure; TEE, transesophageal echocardiography; TMVR, transcatheter mitral valve replacement; TSP, transseptal puncture; TTP-LAAC, thrombus-trapping left atrial appendage closure; ViMAC, valve-in-mitral annular calcification; ViR, valve-in-ring; ViV, valve-in-valve.

## 8. Safety, Complications and Risk Management

TSP is generally considered safe when performed by experienced operators, although it remains an invasive procedure and carries measurable procedural risks. A large multicenter survey involving over 5600 procedures reported an overall complication rate of 1.6%, with cardiac tamponade as the most frequent serious event (0.8%) and a procedure-related mortality rate of 0.02% [34].

Mechanical resistance of the interatrial septum—often due to fibrosis from prior procedures or anatomical variation—poses a technical challenge. When the conventional Brockenbrough technique fails, application of unipolar RF energy through the transseptal needle enables rapid and controlled septal crossing with minimal force. In one series, left atrial access was achieved in all such cases without complications. The number of prior transseptal catheterizations was identified as the sole predictor of septal resistance, likely reflecting cumulative tissue remodeling [27].

Modifications to the standard technique can further reduce procedural risk. For example, advancing a 0.014-inch angioplasty wire through the transseptal needle and positioning it in the left upper pulmonary vein before sheath insertion creates a coaxial rail. This approach enhances control and minimizes trauma to the left atrial wall, particularly in patients with aneurysmal or mobile septa. This wire-assisted method has been performed safely across multiple cases [26].

Certain mechanical injury patterns deserve explicit recognition as procedural pitfalls. Excessive forward pressure on a mobile, aneurysmal, or resistant septum may create pronounced septal tenting before sudden release, producing a “bow-and-arrow” type effect with risk of uncontrolled needle advancement, left atrial wall trauma, posterior wall contact, or pericardial effusion. A related mechanism may occur when initial septal crossing is followed by forceful sheath, balloon, or device manipulation, creating separate septal or atrial injury points rather than a single controlled access track. This pattern, sometimes described as a “double-stitch” injury during balloon-based mitral interventions, reinforces the need for controlled tenting, avoidance of excessive mechanical force, confirmation of needle and wire position before sheath advancement, and early use of TEE or ICE when septal mobility, resistance, or device trajectory is uncertain [26,27,38,39,40,41].

The use of RF-assisted puncture has been validated in prospective studies employing dedicated devices, with reported benefits including high first-pass success and reduced mechanical force on the septum. In these studies, no acute complications such as tamponade or thromboembolism were observed [11].

Another safety-related consideration involves anticoagulation. Maintaining uninterrupted oral anticoagulation during TSP significantly reduces thromboembolic events compared to heparin bridging and does not increase the incidence of major bleeding. This strategy has demonstrated favorable safety outcomes and is now widely adopted in clinical practice [38].

A separate study involving RF-assisted TSP with a dedicated system similarly reported high safety and efficacy, with all procedures completed successfully and no significant complications, further supporting the role of RF energy in overcoming septal resistance without increasing procedural risk [39].

TEE remains indispensable to exclude left atrial thrombus. While the presence of thrombus near the septum or protruding into the atrial cavity typically contraindicates TSP, carefully selected patients with confined, organized thrombi in the left atrial appendage have successfully undergone intervention after a period of intensified anticoagulation [35].

In pregnant patients, TSP has been performed safely during balloon mitral valvuloplasty under echocardiographic guidance, avoiding maternal or fetal complications. Nevertheless, these procedures require meticulous planning and experienced operators [42].

In patients supported by venoarterial extracorporeal membrane oxygenation (VA-ECMO), transseptal access for left atrial decompression adds further procedural complexity. However, when performed under combined TEE and fluoroscopic guidance with careful attention to anticoagulation and anatomy, the procedure can be completed successfully without major complications [6].

Iatrogenic atrial septal defects (iASDs), particularly after transcatheter mitral valve repair, may persist and have been associated with adverse clinical outcomes. In a randomized trial, closure of these defects at one month did not reduce mortality or heart failure hospitalization, although persistent iASD itself was linked to higher overall event rates, suggesting a role for structured follow-up [42].

Rare but serious complications include inadvertent puncture of the aorta. Preventive strategies include careful evaluation of septal tenting, pressure tracing, or contrast injection before sheath advancement, and avoiding excessive force. In one reported case, the ascending aorta was unintentionally cannulated and successfully closed percutaneously using an Amplatzer™ Duct Occluder. Still, reversal of heparin with protamine triggered thrombus formation and coronary embolism, highlighting the risks of routine anticoagulant reversal in the absence of bleeding [43].

Outcomes following aortic puncture vary by location. Conservative management may be sufficient for punctures involving the aortic sinus or sinotubular junction when active bleeding is absent. In contrast, entry into the epicardial space or free wall often results in tamponade and typically necessitates surgical repair. Careful imaging interpretation and real-time confirmation of left atrial access are critical to avoid such complications [41].

Data from over 4600 consecutive TSPs performed at a high-volume center using fluoroscopy and pressure monitoring alone reported a complication rate of 0.72%, including 0.59% for tamponade. Higher complication rates were seen in complex ablations, such as those for atrial fibrillation (0.88%), compared to simpler procedures like accessory pathway ablation (0.17%). Importantly, operator experience had a major influence on outcomes, with complication rates decreasing from 1.3% in early cases to 0.4% after 100 procedures. Adjunctive imaging was used selectively for specific anatomical challenges, supporting its targeted application in expert settings [41].

The available literature does not provide a uniform randomized comparison of fluoroscopy-only versus TEE-guided TSP across all indications. Existing data are heterogeneous and reflect different procedural settings, including routine electrophysiology procedures, balloon mitral valvuloplasty, structural interventions, VA-ECMO decompression, and selected high-risk LAAC cases. Even so, the comparison is clinically useful. Table 5 summarizes the reported performance, complication profile, and practical trade-offs of the two approaches.

These data should not be interpreted as proving universal superiority of one guidance strategy over the other. Rather, they support a risk-adapted approach: fluoroscopy-guided TSP is appropriate for routine cases in experienced centers, whereas TEE guidance is preferred when direct septal visualization, puncture-height control, or early complication detection is required.

In patients with persistent left atrial thrombus who are not suitable for standard anticoagulation or thrombus resolution strategies, a thrombus-trapping approach (TTP-LAAC) has emerged as an alternative. In a multicenter registry of 1918 left atrial appendage closure procedures, 53 patients underwent TTP-LAAC with 100% procedural success. At 30-day follow-up, only 6% developed mild pericardial effusion, with no reported tamponade or embolic events [43].

An overview of major safety-focused studies, their procedural techniques, complication rates, and risk mitigation strategies is presented in Table 6.

## 9. Discussion

The landscape of TSP continues to evolve, driven by innovations aimed at improving safety, procedural efficacy, and reproducibility across a wide spectrum of clinical settings. This review contributes to the growing body of literature by synthesizing key advances in imaging, device technology, and risk mitigation strategies, with particular attention to real-world application in structural heart and electrophysiological interventions. At the same time, the available evidence should be interpreted with caution, because many contemporary technical advances have been evaluated mainly through observational cohorts, registries, technical series, and expert-center experience rather than through randomized comparative studies. This limits the strength of direct comparisons between conventional mechanical puncture, RF-assisted systems, ICE-guided workflows, fusion imaging, and fluoroless strategies. A primary concern during left atrial procedures is the risk of thromboembolic events. In the COMPARE trial, Di Biase et al. [34] demonstrated that maintaining uninterrupted warfarin therapy significantly reduced both stroke and minor bleeding compared to interrupted anticoagulation strategies, particularly in patients with long-standing persistent atrial fibrillation and elevated CHADS_2_ scores. These findings have reinforced the paradigm shift toward maintaining therapeutic anticoagulation throughout TSP-based procedures. However, anticoagulation evidence is not always specific to the transseptal step itself, and outcomes may be influenced by the broader procedural context, patient risk profile, ablation strategy, and periprocedural management protocol. Operator expertise also has an important role in minimizing complications. Simulation-based training has become an increasingly important component of electrophysiology education, helping operators develop procedural skills in a controlled environment before performing transseptal puncture in clinical practice. High-fidelity simulation may be particularly valuable for early-career operators learning complex TSP techniques. This is especially relevant because newer approaches, including ICE-guided TSP, fluoroless navigation, fusion imaging, and RF-assisted puncture, are not purely device-driven solutions. Their safety and reproducibility depend not only on the technology itself but also on operator experience, team coordination, institutional expertise, familiarity with imaging planes, and the ability to recognize unfavorable septal anatomy before puncture.

Technical refinements have further improved transseptal access. Capulzini et al. [35] reported that RF-assisted puncture improves procedural success and reduces complications in patients with challenging septal anatomy. McWilliams and Tchou [11] also demonstrated that applying RF energy through standard transseptal needles provides a low-cost, effective, and safer alternative to excessive mechanical force. Nevertheless, RF-assisted systems should be viewed as adjunctive tools rather than universal replacements for conventional needle-based TSP. Their value is clearest in resistant, thickened, aneurysmal, fibrotic, postsurgical, or previously instrumented septa, whereas routine anatomy can often be managed safely with standard mechanical puncture when performed by experienced operators under appropriate imaging and pressure monitoring. Cost, generator availability, device compatibility, and institutional purchasing constraints may also limit routine adoption in some centers.

In cases involving septal fibrosis or failed access attempts, balloon atrial septoplasty has emerged as a useful bailout strategy, which showed that this approach can restore catheter maneuverability without introducing significant risk. However, repeat puncture or forceful dilation may lead to persistent (iASDs. The clinical implications of iASDs remain under investigation. In the MITHRAS trial, Lurz et al. [37] found no mortality or hospitalization benefit associated with routine closure of iASDs following transcatheter mitral valve repair, advocating for individualized, symptom-based management strategies. These findings also show why procedural success should not be assessed only by immediate sheath passage or device delivery. Long-term septal consequences, residual shunting, right-sided volume loading, and delayed need for closure remain relevant outcomes, especially after large-bore access or repeated septal manipulation.

TSP has also been extended into novel applications beyond its conventional scope. Alkhouli et al. [6] demonstrated the feasibility of using TSP for left ventricular decompression during VA-ECMO. This approach offers a minimally invasive alternative to surgical venting and may be particularly useful in critically ill patients requiring circulatory support. However, evidence in this setting remains limited and highly selected. Critically ill VA-ECMO patients differ substantially in anatomy, hemodynamics, anticoagulation status, bleeding risk, and procedural tolerance, so results from specialized centers should not be generalized without caution.

As procedural complexity increases, preprocedural planning and advanced imaging become indispensable. Russo et al. [40] highlighted the utility of multimodality imaging—including three-dimensional TEE, cardiac CT, and fluoroscopic fusion overlays—in optimizing puncture site selection and reducing complications in patients with altered anatomy or previous interventions. From a critical standpoint, advanced imaging improves anatomical confidence but also introduces practical constraints. CT-based planning requires image acquisition, segmentation, contrast exposure, radiation, and time-sensitive integration with live procedural anatomy. Fusion platforms require dedicated software, trained personnel, and stable imaging-registration workflows. These requirements may restrict their availability outside high-volume structural heart programs.

ICE deserves particular emphasis within this evolving imaging landscape. Its use has increased as electrophysiology and structural laboratories have gained access to dedicated intracardiac imaging catheters and as low-fluoroscopy workflows have become more common. ICE allows direct visualization of the fossa ovalis, septal tenting, needle–sheath contact, and left atrial entry, while avoiding esophageal instrumentation and often allowing the primary operator to control imaging directly. Recent consensus and procedural datasets support its value during TSP, particularly in abnormal atrial size, distorted thoracic anatomy, congenital or postsurgical septal anatomy, redo procedures, and AF ablation workflows aiming to reduce radiation exposure [22,23]. Modified ICE-guided strategies, including workflows using ablation catheter guidance, have also been associated with fewer puncture attempts and shorter puncture duration without compromising procedural success or safety [24]. Nevertheless, these data should be interpreted cautiously, because ICE-guided TSP remains operator- and workflow-dependent, requires additional venous access and training, and may not be equally available across institutions. In addition, many ICE-guided studies come from centers with established electrophysiology imaging expertise, which may overestimate reproducibility in lower-volume laboratories or in teams early in the learning curve.

Persistent iASDs following mitral edge-to-edge repair are increasingly recognized. Chao et al. [41] found no significant differences in mortality or heart failure hospitalization among patients with residual iASDs, suggesting that conservative management may be reasonable in the absence of hemodynamic compromise. Follow-up should nevertheless be individualized, particularly after large-bore transseptal access or repeated septal manipulation. Postprocedural assessment may include transthoracic or transesophageal echocardiography to evaluate residual shunt size, direction of flow, right-sided chamber volume loading, pulmonary pressures, and the presence of bidirectional or right-to-left shunting. Routine closure is not supported for all residual iASDs, but closure may be considered when the defect is associated with clinically relevant hypoxemia, paradoxical embolic risk, significant right-sided volume overload, progressive symptoms, or substantial septal tearing. This approach preserves a conservative strategy for small, hemodynamically silent defects while recognizing that selected patients require structured surveillance and possible delayed intervention [38,41]. Although right femoral venous access remains the standard route for TSP, alternative approaches may offer advantages in selected cases. Maisano et al. [45] reported that left femoral access can provide improved alignment for mitral interventions when right-sided trajectories are suboptimal. While rarely employed, this alternative access route may benefit anatomically challenging cases. Even so, alternative venous trajectories should be interpreted as problem-solving strategies for selected anatomies, not as broadly applicable alternatives. They may alter sheath orientation, reduce familiar tactile feedback, and require careful imaging confirmation before septal crossing.

Redo TSP is another increasingly relevant challenge as more patients undergo repeated left atrial electrophysiology and structural heart procedures. Prior puncture sites, septal fibrosis, surgical patches, baffles, atrial septal occluders, or residual iASDs may alter septal compliance and make standard fossa ovalis engagement more difficult [8,27,44,46]. In these cases, preprocedural CT, TEE, ICE, or fusion imaging may help identify a safe residual septal window and avoid puncture through thickened, scarred, or device-covered tissue. Left femoral venous access may also be considered when the right femoral trajectory is unavailable or provides unfavorable catheter alignment, particularly in selected mitral interventions; however, it may change sheath orientation, reduce familiar tactile feedback, and require modified catheter manipulation and imaging confirmation [45]. These scenarios support an individualized approach in which redo access and nonstandard femoral trajectories are planned according to septal anatomy, device trajectory, and operator experience. Despite these advances, the current evidence base remains uneven. Much of the literature on advanced TSP techniques consists of observational studies, single-center series, registries, technical reports, and expert consensus documents rather than randomized comparisons. This limits the strength of conclusions that can be drawn regarding superiority between conventional mechanical puncture, RF-assisted systems, ICE-guided workflows, fusion imaging, or fluoroless strategies. Reported safety and success rates are also strongly influenced by operator experience, institutional volume, patient selection, and the complexity of the planned intervention. Therefore, favorable outcomes from expert centers should not be interpreted as directly generalizable to all laboratories.

Cost and accessibility are also relevant when interpreting the clinical value of newer technologies. RF-assisted systems, ICE catheters, fusion imaging platforms, CT-based planning software, and advanced electroanatomic mapping systems may improve feasibility in selected complex cases, but they require dedicated infrastructure, training, and institutional resources. In many settings, fluoroscopy-guided TSP with pressure monitoring and selective echocardiographic support remains the practical standard, especially for routine anatomy and experienced operators. The most balanced approach is therefore not to view advanced imaging or energy-based puncture as universal replacements for classical TSP, but as tools that should be matched to procedural complexity, septal anatomy, patient risk, and local expertise.

Despite these advances, the current evidence base remains uneven. Much of the literature on advanced TSP techniques consists of observational studies, single-center series, registries, technical reports, and expert consensus documents rather than randomized comparisons. This limits the strength of conclusions that can be drawn regarding superiority between conventional mechanical puncture, RF-assisted systems, ICE-guided workflows, fusion imaging, or fluoroless strategies. Reported safety and success rates are also strongly influenced by operator experience, institutional volume, patient selection, imaging availability, and the complexity of the planned intervention. Therefore, favorable outcomes from expert centers should be interpreted as evidence of feasibility and procedural usefulness, not as proof of universal generalizability.

Cost and accessibility are also relevant when interpreting the clinical value of newer technologies. RF-assisted systems, ICE catheters, fusion imaging platforms, CT-based planning software, and advanced electroanatomic mapping systems may improve feasibility and procedural confidence in selected complex cases, but they require dedicated infrastructure, training, and institutional resources. In many settings, fluoroscopy-guided TSP with pressure monitoring and selective echocardiographic support remains the practical standard, especially for routine anatomy and experienced operators. The most balanced approach is therefore not to view advanced imaging or energy-based puncture as universal replacements for classical TSP, but as tools that should be matched to procedural complexity, septal anatomy, patient risk, and local expertise.

These limitations also define the main research priorities for the field. Future studies should move beyond feasibility reporting and provide prospective, procedure-specific comparisons of access strategies, using standardized definitions of procedural success, failed puncture, access-related complications, radiation exposure, procedure time, cost, and long-term sequelae such as persistent iASDs. Multicenter registries and pragmatic comparative studies would be particularly useful for identifying which patients benefit most from advanced imaging-guided or RF-assisted approaches.

Looking forward, future research should focus on developing standardized protocols for complex TSP scenarios, defining long-term outcomes for nontraditional access routes, and establishing evidence-based criteria for iASD closure. Prospective registries and imaging-integrated clinical trials will be instrumental in guiding procedural decision-making and refining best practices.

## 10. Strengths and Limitations

This review offers a clinically oriented synthesis of the anatomical, technical, and clinical dimensions of transseptal puncture, integrating both historical context and recent innovations. A key strength is its dual focus on established practice and emerging technologies, providing actionable insights for both experienced practitioners and those in training. Additionally, its inclusion of safety strategies, procedural techniques, and practical decision-making frameworks enhances its clinical utility. Nonetheless, the review is subject to limitations inherent to its narrative design. Although the literature search and selection process were described to improve transparency, this review was not designed as a systematic review or comparative effectiveness analysis. The absence of formal risk-of-bias assessment, quantitative evidence grading, and meta-analysis introduces potential selection bias. In addition, many of the emerging transseptal technologies discussed in this review, including RF-assisted puncture, ICE-guided workflows, fusion imaging, balloon septoplasty, and fluoroless approaches, are supported mainly by observational studies, registries, technical reports, single-center experience, and expert consensus documents rather than randomized comparative trials.

This limitation is relevant when interpreting reported success and complication rates, which may be influenced by operator experience, institutional volume, imaging availability, patient selection, septal anatomy, and the complexity of the planned intervention. Therefore, the review should be read as a practical synthesis of current techniques, indications, and safety considerations, rather than as evidence that one transseptal access strategy is universally superior to another. Furthermore, the rapid pace of technological development in catheter-based interventions may outstrip the currently available evidence, rendering some aspects prone to obsolescence. Future prospective registries, systematic reviews, and randomized trials will be essential to validate the efficacy and safety of emerging tools and procedural adaptations highlighted in this work.

## 11. Conclusions

This TSP has evolved from a purely diagnostic technique into a cornerstone of contemporary structural and electrophysiological cardiac interventions. Continuous refinements in mechanical tools, radiofrequency-assisted systems, and imaging guidance have enabled safer, more precise, and more reproducible access to the left atrium across a broad range of clinical contexts. The integration of advanced imaging modalities, steerable sheaths, and energy-based puncture devices has significantly enhanced procedural outcomes, particularly in patients with challenging or altered septal anatomy.

Today, the clinical applications of TSP extend well beyond traditional diagnostic and ablation procedures. The technique is fundamental to advanced interventions such as transcatheter mitral valve repair, left atrial appendage occlusion, and ventricular decompression in patients supported by ECMO. Adjunctive strategies—including balloon septoplasty, thrombus-trapping closure systems, and fluoroscopy-free navigation—further broaden the procedural toolkit. Across large multicenter studies, TSP has demonstrated a favorable safety profile when performed by experienced operators, with radiofrequency-enabled systems and uninterrupted anticoagulation emerging as important tools in risk reduction.

## Figures and Tables

**Table 1:** Comparative overview of imaging modalities used for transseptal access.

Imaging Modality	Main Procedural Role	Main Advantages	Main Limitations	Preferred Settings
Fluoroscopy ± pressure monitoring	Tracks catheter position, pull-down, sheath movement, and RAO/LAO orientation	Widely available; familiar; rapid spatial orientation	Indirect anatomy; no direct visualization of septal tissue, fossa ovalis, or tenting depth	Standard anatomy; routine EP procedures; experienced centers
TEE	Defines fossa ovalis, septal tenting, puncture height, anterior–posterior orientation, and early complications	Detailed soft-tissue imaging; strong for structural procedures and device alignment	Requires echocardiographer; may require deep sedation/general anesthesia; esophageal instrumentation	TEER/MitraClip, TMVR, LAAC, VA-ECMO decompression, complex structural procedures
ICE	Provides intracardiac visualization of septal anatomy, needle/sheath contact, tenting, and LA entry	Operator-controlled; useful in EP workflows; avoids esophageal instrumentation; may reduce fluoroscopy; compatible with 3D mapping	Additional venous catheter; cost; learning curve; image-plane dependence	AF ablation; redo EP procedures; abnormal septal anatomy; selected LAAC/structural cases; low- or zero-fluoroscopy workflows
MSCT/cardiac CT	Maps septal and LA anatomy before the procedure	Patient-specific planning; supports trajectory and device-alignment assessment	Not inherently real-time; radiation and contrast exposure	LAAC, TMVR/TEER planning, altered anatomy, prior septal intervention
Fusion imaging	Integrates live fluoroscopy with TEE or CT-derived anatomy	Improves spatial orientation and puncture-site planning in complex cases	Requires dedicated software, infrastructure, and team experience	Structural heart interventions; repeat procedures; complex anatomy
Electroanatomic/fluoroless guidance	Integrates catheter tracking with 3D chamber geometry and septal localization	Reduces radiation; fits EP workflows; can be combined with ICE	Operator- and system-dependent; limited to experienced centers	AF ablation and other left-sided EP procedures using established low- or zero-fluoroscopy protocols

Abbreviations: AF, atrial fibrillation; CT, computed tomography; EP, electrophysiology; ICE, intracardiac echocardiography; LA, left atrium; LAAC, left atrial appendage closure; LAO, left anterior oblique; MSCT, multislice computed tomography; RAO, right anterior oblique; TEE, transesophageal echocardiography; TEER, transcatheter edge-to-edge repair; TMVR, transcatheter mitral valve replacement; VA-ECMO, venoarterial extracorporeal membrane oxygenation.

**Table 2:** Comparative Summary of Transseptal Puncture Tools and Techniques.

Tool/Technique	Mechanism	Advantages	Limitations	Clinical Use Case
Brockenbrough Needle	Mechanical puncture	Widely available; cost-effective	Requires significant force in fibrotic or aneurysmal septa	First-line method in standard anatomy
RF-Assisted Needle (e.g., VersaCross)	Low-energy radiofrequency puncture	Facilitates smooth septal crossing; high success in resistant tissue	Needs dedicated RF system; higher cost	Fibrotic, thickened, or post-ablation septa
SafeSept Guidewire	Sharp-tipped guidewire with flexible shaft	Minimizes perforation risk; tracks well with standard sheaths	Limited steerability; lacks support in tortuous anatomy	Difficult anatomy; minimizes trauma
Balloon Septoplasty	Mechanical dilation using noncompliant balloon	Effective bailout for resistant septa; restores sheath passage	Balloon trauma risk; not routine first-line	Failed standard or RF puncture; prior patch/device crossing
Electrocautery-Modified Wire	Electrosurgical puncture via standard wire	Adaptable with standard EP lab equipment; off-label but effective	Operator-dependent; lacks precise energy control	Backup method when needle access fails
Fusion Imaging Guidance	CT or TEE overlay onto fluoroscopy	Enhances precision; improves spatial orientation; reduces complications	Requires imaging software/infrastructure; adds procedural planning	High-risk anatomy; mitral and appendage interventions

**Table 5:** Practical comparison of fluoroscopy-guided and TEE-guided transseptal puncture based on available reported data.

Guidance Strategy	Reported Procedural Performance	Reported Complications/Safety Findings	Main Advantages	Main Limitations
Fluoroscopy ± pressure monitoring	High procedural feasibility in experienced EP centers; large high-volume series support routine use in standard anatomy [32,42]	Overall complication rate 1.6% in a large multicenter EP survey, with tamponade reported in 0.8% [32]; fluoroscopy-only high-volume data reported 0.72% overall complications and 0.59% tamponade [42]	Widely available; rapid; familiar to operators; does not require continuous echocardiographic imaging; efficient for routine EP workflows	Indirect anatomical guidance; no direct visualization of fossa ovalis, septal tenting, or adjacent structures; outcomes strongly influenced by operator experience; less informative in distorted septal anatomy or complex structural procedures
TEE-guided TSP	Used successfully in structural and high-risk settings, including balloon mitral valvuloplasty in pregnancy, VA-ECMO left atrial decompression, and selected LAAC scenarios [6,35,36,43]	Reported safety is favorable in selected series, including no maternal or fetal complications in pregnancy cases [36], no major complications in VA-ECMO decompression cases [6], and 100% procedural success with 6% mild pericardial effusion and no reported tamponade or embolic events in selected TTP-LAAC patients [43]	Direct visualization of septal anatomy, tenting, puncture height, LA entry, and early complications; particularly useful for TEER/MitraClip, TMVR, LAAC, VA-ECMO decompression, and complex anatomy	Requires echocardiographic expertise; may require deep sedation or general anesthesia; involves esophageal instrumentation; adds logistical and procedural complexity

Abbreviations: EP, electrophysiology; LA, left atrium; LAAC, left atrial appendage closure; TEE, transesophageal echocardiography; TEER, transcatheter edge-to-edge repair; TMVR, transcatheter mitral valve replacement; TSP, transseptal puncture; TTP-LAAC, thrombus-trapping left atrial appendage closure; VA-ECMO, venoarterial extracorporeal membrane oxygenation.

**Table 6:** Overview of Safety Strategies and Complication Rates in Transseptal Puncture.

Study	Study Context	Sample Size	Guidance Used	Technique Description	Complication Rate	Risk Mitigation Strategy
Hildick-Smith et al. [44]	Wire-assisted technique	30	Fluoroscopy + wire	Angioplasty wire advanced through needle	0%	Pulmonary vein anchoring to avoid wall trauma
Knecht et al. [26]	Resistant septa	269	RF with fluoroscopy	RF-assisted puncture via ablation catheter	~0.4%	Avoided mechanical force by using RF
De Ponti et al. [32]	Large EP center survey	5660	Mixed	Standard TSP	1.6%	Operator experience significantly reduced complications
Di Biase et al. [34]	Anticoagulation strategy	COMPARE trial	Standard	TSP under uninterrupted warfarin	↓ Stroke, ↓ Bleeding	Continued anticoagulation peri-procedure
Capulzini et al. [35]	RF puncture device	104	Fluoro + RF system	Dedicated RF needle system	0%	Controlled septal entry, no mechanical force
Gamra et al. [36]	TSP in pregnancy	Not specified	TEE	Balloon mitral valvuloplasty	0%	Echo-only approach, avoided radiation
Lurz et al. [37]	iASD after MitraClip	RCT	Post-procedure TEE	Conservative vs. closure strategy	Higher with iASD	Structured follow-up for iASD patients
Pravda et al. [38]	Aortic perforation	Case report	Fluoroscopy	Misplacement of sheath into aorta	STEMI	Avoided protamine unless active bleeding
Chen et al. [39]	Aortic root puncture	27 cases	Fluoroscopy	Unintentional puncture of sinus or root	Site-dependent	Confirm anatomy, use pressure/contrast before sheath advance
Matoshvili et al. [42]	High-volume EP center	4690	Fluoro only	Standard TSP	0.72% (0.59% tamponade)	Improved with operator experience; selective imaging use
Sebag et al. [43]	LA thrombus cases (TTP-LAAC)	53/1918 total	TEE	Thrombus-trapping LAAC procedure	6% mild effusion	Avoided protamine; thrombus containment via device
Alkhouli et al. [6]	TSP during VA-ECMO	Case series	TEE + fluoroscopy	Left atrial decompression (venting)	None reported	Multidisciplinary planning; real-time imaging guidance

Abbreviations: EP, electrophysiology; iASD, iatrogenic atrial septal defect; LA, left atrium; LAAC, left atrial appendage closure; RCT, randomized controlled trial; RF, radiofrequency; STEMI, ST-segment elevation myocardial infarction; TEE, transesophageal echocardiography; TSP, transseptal puncture; TTP-LAAC, thrombus-trapping left atrial appendage closure; VA-ECMO, venoarterial extracorporeal membrane oxygenation; ↓-decreased.

## Data Availability

No new data were created or analyzed in this study. Data sharing is not applicable to this article.

## Abbreviations

ABS	Atrial Balloon Septoplasty
AF	Atrial Fibrillation
CT	Computed Tomography
EP	Electrophysiology
iASD	Iatrogenic Atrial Septal Defect
LAAC	Left Atrial Appendage Closure
MSCT	Multislice Computed Tomography
RF	Radiofrequency
STEMI	ST-Elevation Myocardial Infarction
TEE	Transesophageal Echocardiography
TP	Transseptal Puncture
TTP-LAAC	Thrombus-Trapping Left Atrial Appendage Closure
TMVR	Transcatheter Mitral Valve Replacement
VA-ECMO	Veno-Arterial Extracorporeal Membrane Oxygenation
ViMAC	Valve-in-Mitral Annular Calcification
ViR	Valve-in-Ring
ViV	Valve-in-Valve
